# Superior lumbar hernia after gastrectomy repaired via an open approach in the prone position: A case report

**DOI:** 10.1016/j.ijscr.2020.05.046

**Published:** 2020-05-29

**Authors:** Yasutaka Nakanishi, Yasunori Kurahashi, Yoshinori Ishida, Mitsuru Sasako, Hisashi Shinohara

**Affiliations:** Department of Surgery, Upper Gastrointestinal Division, Hyogo College of Medicine, Japan

**Keywords:** Hernioplasty, Lumbar hernia, Prone position, Underlay mesh, Case report

## Abstract

**Introduction:**

Lumbar hernia is a rare hernia in the posterolateral abdominal wall and only about 310 cases are known to have been reported to date. Laparoscopic hernioplasty is a common surgical approach but is unsuitable for patients who have previously undergone laparotomy and are expected to have extensive visceral adhesions.

**Presentation of Case:**

An 84-year-old woman who had undergone an open distal gastrectomy was referred to our hospital with an enlarging but easily reducible bulge in the right upper back. On computed tomography, the hernial orifice was located in the lateral side of the right quadratus lumborum under the costal arch. The bulge was diagnosed as a superior lumbar hernia. We performed an open hernioplasty in the prone position to avoid internal visceral adhesions. The hernia sac was detected in the latissimus dorsi in the back, and was found to contain the ileocecum, which was rigidly adherent to the sac. Hernioplasty was performed by inserting polypropylene mesh between Zuckerkandl’s fascia and the internal oblique.

**Discussion:**

Mechanical ileus after open distal gastrectomy is common complication and sometimes position was simple procedure without the influence of visceral adhesion and easily reinforced by underlay mesh.

**Conclusions:**

Open hernioplasty in the prone position using a mesh underlay is an optional approach in a patient with a superior lumbar hernia after gastrectomy.

## Highlights

• Lumbar hernia is a rare hernia occurring in the posterolateral abdominal wall and
• suitable for laparoscopic hernioplasty.
• Intraabdominal approach is sometimes difficult for superior lumber hernia after gastrectomy with expected visceral adhesions.
• Open hernioplasty with underlay mesh in prone position is an optional approach to avoid internal visceral adhesion.
• Mesh must be set between Zuckerkandl’s fascia and internal oblique to avoid prolapse of abdominal and retroperitoneal organs.

## 1 Introduction

**Fig. 1:**
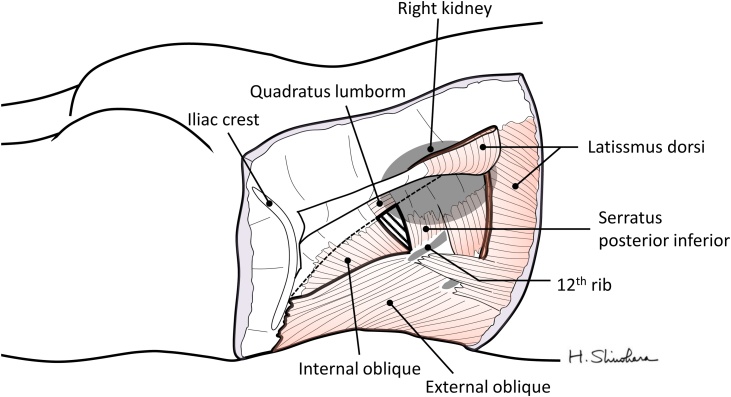
Schematic of the right superior lumbar space in a patient with a superior lumbar hernia. The triangular area outlined by dotted lines indicates the hernial orifice.

Lumbar hernia is a rare hernia of the posterior abdominal wall, with only about 310 cases reported worldwide [1,2]. The orifice is usually located in the superior or inferior lumbar space. The superior lumbar space consists of the costal arch, the internal oblique, and the quadratus lumborum (Fig. 1), while the inferior lumbar space consists of the iliac crest, the external oblique, and the latissimus dorsi [1].

Ravaton first reported using herniorrhaphy to treat a strangulated lumbar hernia in a pregnant woman in 1750 [1]. Since the 1950s, tension-free mesh repair has replaced primary closure as the first choice for reinforcement [3,4]. Laparoscopic hernioplasty was introduced in 1990, and has become the standard from the perspective of a good visual angle and minimal invasiveness [[5], [6], [7]]. However, no operative procedures have been established for this rare type of hernia, probably because of the limited opportunities for surgical repair of this condition. Furthermore, a laparoscopic approach may be unsuitable for patients who have previously undergone laparotomy and are likely to have extensive visceral adhesions.

Here, we report on a patient with a superior lumbar hernia after gastrectomy and demonstrate that open hernioplasty using mesh in the prone position may be an optimal approach in a patient with a history of laparotomy. This case report was reported with SCARE criteria [8].

## 2 Case presentation

**Fig. 2:**
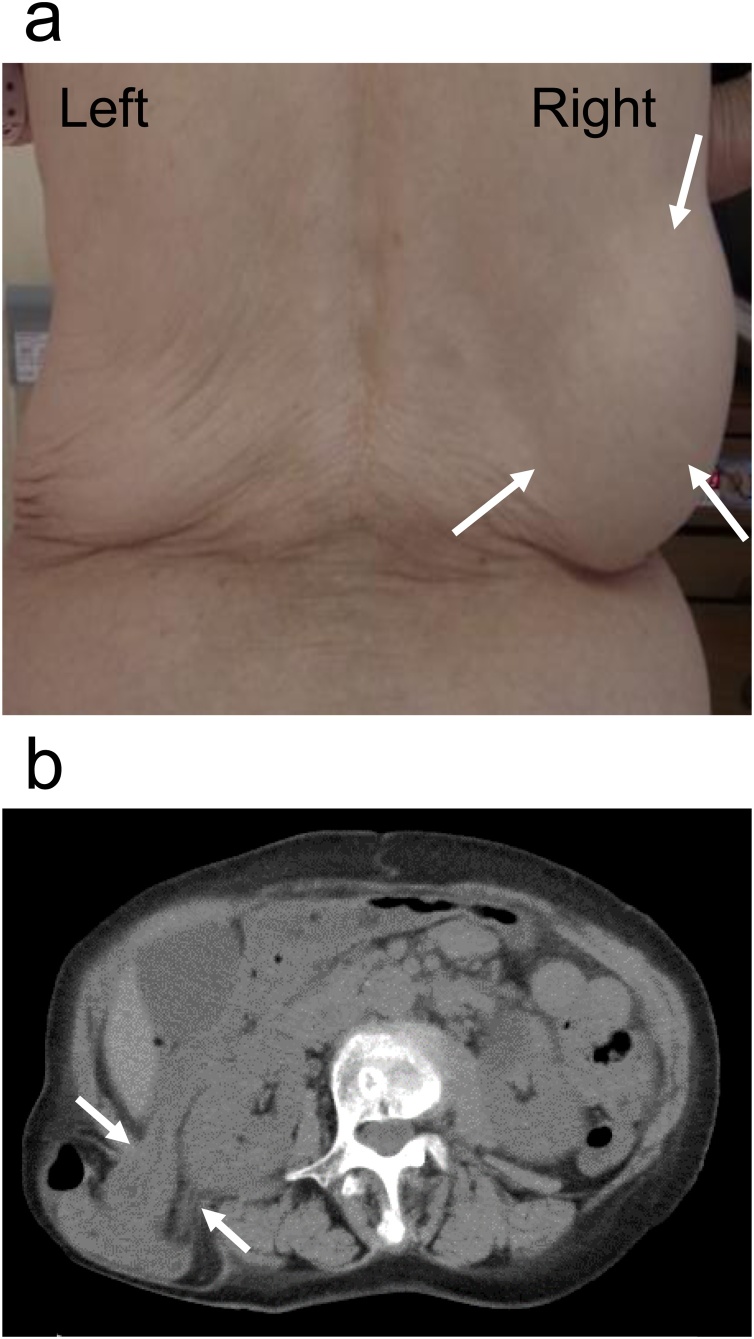
Physical and radiologic images of the right superior lumber hernia. (**a**) Physical findings in the standing position. (**b**) Computed tomography imaging showing right superior lumbar hernia. (**a**) A reducible bulge in the right upper back can be seen (arrows). (**b**) Herniation in the right superior lumbar space. The hernia is assumed to contain the ascending colon (arrowheads).

An 84-year-old woman presented to our hospital for follow-up of a distal gastrectomy and Roux-enY reconstruction for gastric cancer and was found to have a right lumbar hernia. The patient had noticed an enlarging bulge in mid aspect of the right back, which was about the size of a tennis ball but was easily reducible (Fig. 2a). Computed tomography revealed the hernial orifice to be on the lateral side of the right quadratus lumborum just causal to the costal arch (Fig. 2b). The thickness of the right transversus abdominis and quadratus lumborum was decreased compared with that on the left. The hernia appeared to contain the ascending colon. The right kidney was seen to be sliding towards the hernial orifice.

**Fig. 3:**
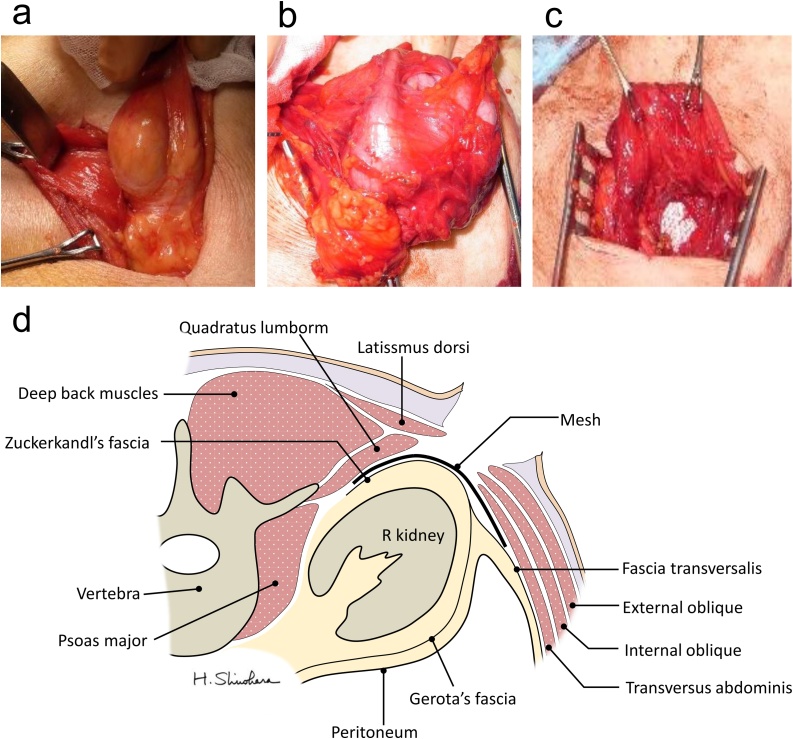
Intraoperative findings and surgical approach to repair the right superior lumber hernia. (**a**-**c**) Intraoperative findings and (**d**) Schema of open approach hernioplasty for right superior lumbar hernia indicating the location of the underlay mesh inserted. (**a**) The hernia sac is identified within the latissimus dorsi on the back. (**b**) Ileal and cecal tissue contained in the hernia is adherent to the hernia sac. (**c, d**) A mesh underlay is inserted between Zuckerkandl’s fascia and preperitoneal fascia.

We opted to use an open approach for the surgical repair to avoid laparotomy because of the visceral adhesions expected after the distal gastrectomy. The patient was placed in the prone position and a 7-cm oblique incision was made over the hernial orifice. The latissimus dorsi was then mobilized from the lateral side, and the hernia sac was detected in the posterior aspect of the muscle (Fig. 3a). The content of the hernia was the ileocecum, which was extensively adherent to the hernia sac (Fig. 3b). The diameter of medial side of the orifice, exposing the right kidney. After repositioning the hernia sac and its contents within the abdominal cavity, polypropylene mesh (Ventrio™ Small Oval Hernia Patch, Bard Medical, Covington, GA) was inserted between the internal oblique and Zuckerkandl’s fascia to prevent herniation of the preperitoneal organs (Fig. 3c, d); the mesh was fixed quadratus lumborm using two sutures to avoid dislocation.

The postoperative course was uneventful and the patient was discharged 7 days after surgery. There has been no recurrence during 2 years of follow-up.

## 3 Discussion

The first case report of a lumbar hernia was by Garangeot in 1731. The anatomic details of the superior lumbar space around the hernial orifice were described by Grynfeltt and those of the inferior lumbar space by Petit [1]. Two etiopathogenetic mechanisms for lumbar hernia have been proposed, namely congenital and acquired. Congenital lumbar hernia is usually encountered in association with other congenital malformations, and has been reported in nearly 50 cases [9], whereas acquired lumbar hernia typically results from trauma, surgical incision, or an abdominal abscess, as well as spontaneously. Blunt abdominal trauma associated with a crush injury or a motor vehicle accident has been

repair of an abdominal aorta aneurysm, resection of an abdominal wall tumor, iliac bone graft, and a latissimus dorsi flap procedure are well-known iatrogenic causes of lumbar hernia [1,10]. Aging, loss of body weight, chronic disease, postoperative sepsis, and muscle atrophy are all known risk factors for spontaneous lumbar hernia. Our patient was an elderly woman whose lumbar hernia could have been spontaneous and it may have already been present at the time of the gastrectomy for early stomach cancer. Aging and body weight loss may affect the enlargement of lumber hernia.

The contents of a lumbar hernia may include the small intestine, colon, part of the liver, the stomach, omentum, retroperitoneal fat, ovary, spleen, and kidney [11]. Incarceration is rare, but obstruction of the intestinal or urinary tract can occur if the hernia includes any of these organs. Computed tomography reliably estimates the size, location, and contents of the hernia, and provides useful information about skeletal muscle atrophy around the hernial orifice.

Mechanical ileus after open distal gastrectomy is common complication. Kawamura et al. reported that adhesive small bowel obstruction requiring operation arose in 5.7 percent of the patients undergone open distal gastrectomy [12]. Additionally, omentectomy and dissection of the duodenal bulb more often induced severe adhesion observation of the orifice in right upper lumber space by laparotomy.

Several surgical approaches can be used to repair a lumbar hernia. Tension-free mesh hernioplasty is more effective than conventional herniorrhaphy in patients with associated muscle atrophy [1]. Laparoscopic hernioplasty using mesh was recently been reported to be superior to open hernioplasty because of lower postoperative morbidity, short operating time, and shorter hospital stay [7]. However, laparoscopic repair would be difficult to perform and potentially unsuccessful in patients with extensive abdominal adhesions. Our patient had a small-sized hernial orifice, but was expected to have visceral adhesions after previous gastrectomy. Therefore, we considered that an open approach using a mesh underlay was optional method to allow to avoid laparotomy. In this type of repair, it is important to insert the mesh under Zuckerkandl’s fascia to prevent prolapse of retroperitoneal tissue. Adequate dissociation is also required to avoid dislocation of the mesh.

Prone position provides surgical access to the dorsal aspects, however, it is associated with ocular and cardiovascular complications [13,14]. Generally, lateral position or prone position were used in the operation of lumber hernia. We considered that open approach in the prone position allowed to exfoliate Zuckerkandl’s fascia directly, lateral position. The hernioplasty in prone position is suitable procedure under the appropriate general anesthesia.

## 4 Conclusion

Open hernioplasty with placement of mesh underlay in the prone position is a simple and adequate procedure in a patient with a superior lumbar hernia who is expected to have visceral adhesions.

## Declaration of Competing Interest

All authors declare that there is no conflict of interest related with this work.

## Sources of funding

The authors declare that no funding was received for this study.

## Ethics approval

This case report study was carried out respecting the Declaration of Helsinki in its current version. Case report is exempt from ethnical approval in our institutional ethnical approval.

## Consent

We obtained the patient’s written informed consent about the publication of this case report and accompany images.

## Authors’ contributions

YN acquired the physical, radiological, and surgical data and drafted the manuscript. MS, YN, and YK performed the surgery. YK, YI, MS, and HS contributed to final revision of the manuscript. The anatomic and surgical images were drawn by HS. HS supervised the case study. All authors read and approved the final manuscript.

## Registration of research studies

1 Name of the registry: Superior lumbar hernia after gastrectomy repaired via an open approach in the prone position
2 Unique identifying number or registration ID:5614
3 Hyperlink to your specific registration (must be publicly accessible and will be checked): https://www.researchregistry.com/browse-the-registry#home/registrationdetails/5ec1da1f9441a60015b66786/

## Guarantor

The Guarantor of this work is Hisashi Shinohara M.D. Ph.D.

## Provenance and peer review

Editorially reviewed, not externally peer-review.
